# Artisanal fishing supports breeding of malaria mosquitoes in Western Kenya

**DOI:** 10.1186/s12936-019-2708-z

**Published:** 2019-03-12

**Authors:** Wolfgang Richard Mukabana, Janet Achieng Onyango, Collins Kalwale Mweresa

**Affiliations:** 1 School of Biological Sciences, University of Nairobi, P.O. Box 30197–00100, Nairobi, Kenya; 2 Science for Health, P.O. Box 44970–00100, Nairobi, Kenya; 3 School of Biological and Physical Sciences, Jaramogi Oginga Odinga University of Science and Technology, P.O. Box 210–40601, Bondo, Kenya

**Keywords:** Artisanal capture fishing, Mosquito larvae, *Anopheles gambiae*, Larval productivity, Habitat, Fishing boats, Malaria, Mageta Island, Kenya, Lake Victoria, Ecosystem health, Ecohealth

## Abstract

**Background:**

Everyday hundreds of people, mainly men, set out to take part in a vibrant artisanal capture fishing (ACF) industry on Lake Victoria. It is not known whether actions of artisanal fishers, in their unrelenting quest for existence, surpass ecosystems’ sustainability thresholds with potentially negative repercussions on human health with respect to malaria transmission potential. This article sought to fill this information gap.

**Methods:**

This study used an ecosystem approach to find out how ACF processes facilitate the breeding of mosquitoes. The observational study adopted a cross-sectional design and was carried out on Mageta Island situated inside Lake Victoria in western Kenya.

**Results:**

Of the 87 mosquito larval habitats identified 27 (31%) were created through ACF activities. The ACF-related habitats, hereafter collectively referred to as ‘fishing habitats’, included fishing boats (24), trenches (1) and fish bait mines (2). About half (48%) of *Anopheles* larvae were recovered from fishing habitats. The mean larval density in the fishing habitats (35.7 ± 1.15) was double that in non-fishing habitats (17.4 ± 0.539). Despite being the most common ‘non-fishing habitat’ type (N = 32), the mean number of *Anopheles* larvae present in rock pools (30.81 ± 10.54) was significantly less than those found inside fishing boats (N = 24; 40.08 ± 10.16). Overall, man-made habitats and those used to support livelihoods contained significantly more *Anopheles* larvae.

**Conclusions:**

These data show that artisanal capture fishing is a key driver of malaria epidemiology on Mageta Island. This suggests that larval source management strategies in the global south should pay attention to the heterogeneity in *Anopheles* breeding habitats created through livelihood activities.

## Background

Everyday hundreds of people, mainly men, depart from home to participate in a vibrant artisanal capture fishing industry in Kenya’s Lake Victoria fishery [1–3]. This industry forms the primary income source for the locals [1, 4–6]. Landed fish is hardly consumed within the fishers’ households [7] due to the small stock sizes [8]. The fish is sold off to generate cash income that is used to buy food, pay for medical care and other basic needs [2, 7]. The proceeds are often supplemented with agricultural produce [1, 9, 10]. Fishing crew must increase effort to find, catch and obtain sizeable stocks from the declining fishery. They cope by migrating to adjacent fisheries perceived to harbor larger fish stocks [1, 11], migrating to fisheries near large economic markets [11, 12], using more extractive fishing gears [1, 13] and use of more effective fishing baits [14].

The evolving and current threat of outdoor transmitted malaria [15, 16], especially in outdoor groups engaging in compelling social, cultural and economic activities at night [17] e.g. capture fishers [18], can be viewed as an ecological disaster [19]. Artisanal capture fishers exert big pressure on the environment [20] through relentless exploitation of fishery resources [3, 21]. As noted elsewhere ‘*poor people are forced to overuse environmental resources to survive from day to day, and their impoverishment of the environment further impoverishes them, making their survival ever more difficult and uncertain’* [22]. Persistent pressure by poverty on ecosystems also has negative repercussions on human health [19, 23–25]. Thus, artisanal capture fishing, as practiced in the Lake Victoria fishery, is not a sustainable livelihood source [1].

In this study, an ecosystem approach [19, 23–25] was employed to understand the association between artisanal fishing and the problem of malaria on Mageta Island in western Kenya. The central goal was to establish whether actions of artisanal fishers, in their unrelenting quest for existence, surpass ecosystems’ sustainability thresholds with potentially negative repercussions on human health. This was achieved through a cross-sectional survey seeking to (a) determine if artisanal capture fishing leads to creation, hence occurrence, of *Anopheles* breeding habitats, and (b) establish the potential correlation between artisanal capture fishing and *Anopheles* larval productivity. In the context of this study artisanal capture fishing is defined as a small-scale activity in which fish are caught in the wild using rudimentary methods. Although some authors have pointed out at the increased risk of malaria in the context of artisanal fishing [18, 26–30], no one has specifically assessed the link between artisanal fishing, habitat degradation and larval ecology of malaria vectors. This is the remit of this article.

## Methods

### Study area

**Fig. 1:**
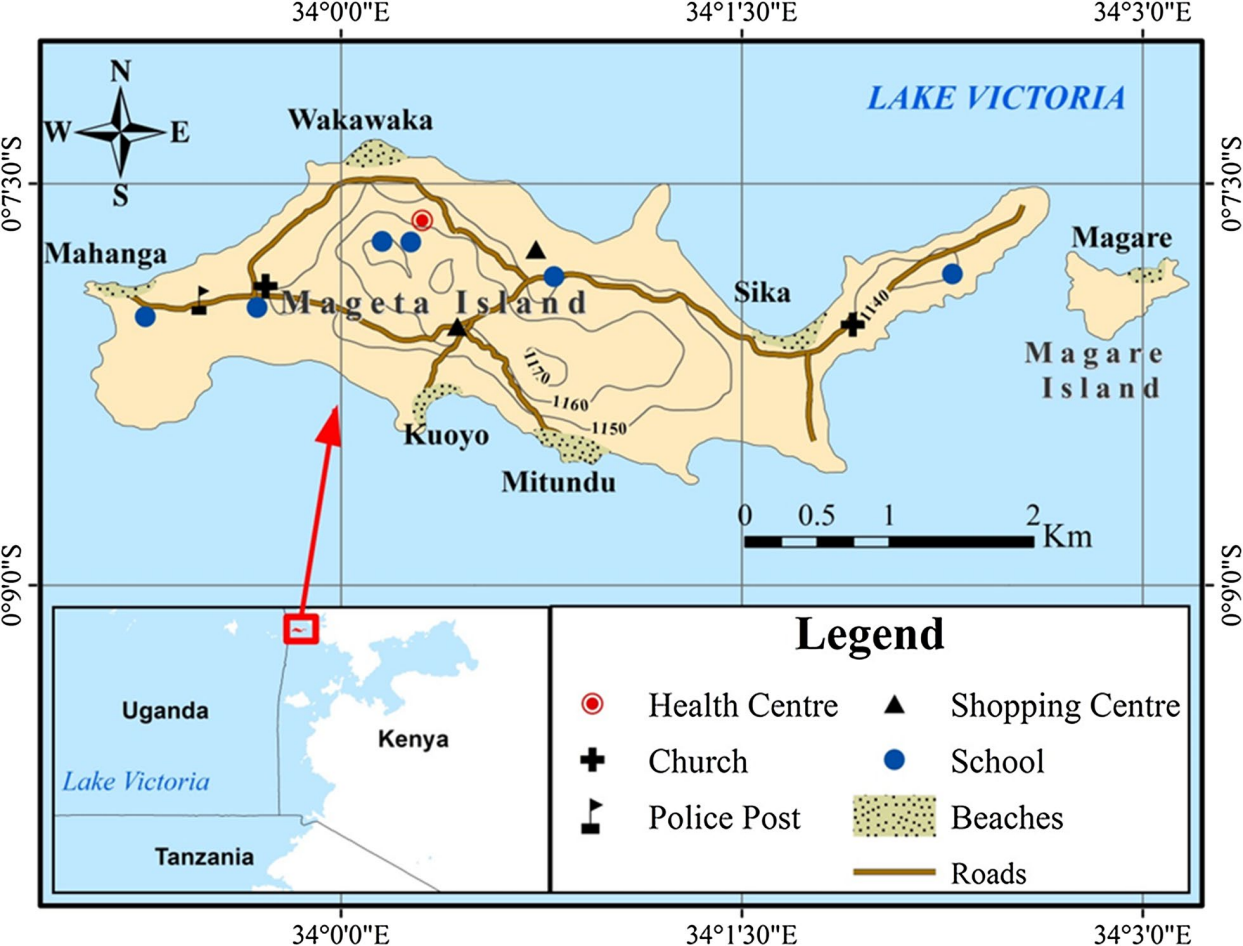
Study area map showing location of Mageta Island in Western Kenya

This study was carried out on Mageta Island located inside Lake Victoria (Fig. 1) in Siaya County in Western Kenya. Mageta lies very close to the Kenya-Uganda border. Administratively, Mageta Island, the adjacent Magare Island and the uninhabited Sirigombe Island form Mageta Location. Mageta Location has a population of approximately 7000 persons and is adjacent to the islands of Wayasi, Siamulala, Hama, Siro and Lolwe in Uganda. Mageta Island lies at an altitude of 1,140 m above sea level between 33° 59′15″–34° 2′30″ E and 0° 7′15″–0°8′15″ N [31]. The surface area of the Island is approximately 7.02 sq km [32].

Mageta location has six fish landing beaches namely Kuoyo, Magare, Mahanga, Mitundu, Sika and Wakawaka. The main fish species caught around Mageta include the Nile tilapia (*Oreochromis niloticus*), the Nile perch (*Lates niloticus*) and the silver cyprinid (*Rastrioneobola argentae*). Other common fishes include species of *Haplochromis*, *Mormyrus* and *Protopterus*. Fresh water shrimps (*Caridina nilotica*) and crabs are also common. These fish and crustaceans are caught using rudimentary methods in a locally active artisanal fishing industry, which constitutes the main livelihood activity for the local inhabitants.

Animal husbandry and small scale crop farming are also practiced on Mageta Island. Domesticated animals include cattle, cats, chicken, dogs, donkeys, geese, goats, pigs and sheep [1]. Wild animals include crocodiles, lizards, monkeys, species of otter, snakes and night-grazing hippopotamuses. Lake flies, snails and many different avian species abound. Crop agriculture is concentrated mainly on the island’s muddy, northern shores. The dominant crop plants include maize, beans, tomatoes and collard greens (*Brassica oleracea*) of the cultivar *Acephala* [1]. Tangerines are also present. The animals and plants most probably serve as blood and sugar meal sources for local mosquito species, respectively.

The main species of *Anopheles* on Mageta Island include *Anopheles gambiae, Anopheles arabiensis, Anopheles funestus* and *Anopheles coustani* [32]. *Plasmodium falciparum* is the main malaria parasite on the Island (pers. comm., Mageta Health Centre). *Anopheles gambiae* and *An. funestus* prefer to blood-feed on humans rather than animal hosts [33]. They also feed at night and rest indoors [33]. Unfortunately, the readily available malaria protection measures, namely long-lasting insecticidal nets, target only indoor, night-biting/resting mosquitoes. Thus, persons engaging in outdoor socioeconomic activities at night, e.g. the artisanal capture fishers of Mageta Island, remain unprotected [34]. This protection gap, together with increased resistance by malaria mosquitoes and parasites to hitherto effective insecticides and drugs [35], respectively, escalates the vulnerability of local inhabitants of Mageta Island to malaria. Innovative, locally sustainable mitigating approaches are urgently needed. In the context of this study the term Mageta Island is generally used to refer to both Mageta and Magare Islands. No work was done on Sirigombe Island.

### Artisanal capture fishing and creation of *Anopheles* larval habitats

An observational study was performed to find out if activities associated with artisanal capture fishing facilitate creation of stagnant water bodies suitable for breeding of *Anopheles* larvae. Potential interaction was measured by determining the probability of finding *Anopheles* larvae in habitats created through artisanal capture fishing. All mosquito larval habitats on Mageta Island were identified, ecologically characterized and mapped through a cross-sectional survey. This analytical approach was effected using community health volunteers, hereafter referred to as community actors, as liaison.

The community actors helped to identify and locate stagnant water bodies where *Anopheles* larvae may have lived. The search focused on identifying all holes, depressions, grooves, furrows, gutters and all earthen, wooden or other containers that held stagnant water. The putative mosquito larval habitats were accessed by walking from one to the next. Guidance was provided by a different community actor [36] in each one of the 22 villages in Mageta Location. Characterization of habitats involved engaging community actors in ecological dialogue [37] that helped to succinctly describe individual habitat types. The discussions focused on how and why the habitats were created, even if inadvertently.

Habitat types created through artisanal capture fishing activities or otherwise were respectively classified as ‘fishing habitats’ or ‘non-fishing habitats’. The outcome of finding (‘*Anopheles* present’) or not finding malaria mosquito larvae (‘*Anopheles* absent’) inside the habitats was determined and recorded. Analysis of the correlation between habitat type as a predictor of *Anopheles* presence in the habitats was used to fill up the research gap. Larval sampling was performed using a 350 ml WHO dipper. Up to ten dips were taken from each larval habitat and inspected before a decision on presence (or absence) of *Anopheles* larvae in individual habitats was reached. Mapping of individual mosquito larval habitats was done with the aid of a GPS receiver (Garmin eTrex^®^ 10). The GPS data points were transferred into ArcGIS software (10.2.2) and used to develop a spot map.

### Artisanal capture fishing and *Anopheles* larval productivity

The effect of artisanal capture fishing on *Anopheles* larval productivity on Mageta Island was evaluated as an integral part of the survey. Causal effects were measured by determining the statistical relationship between habitat type (i.e. ‘fishing’ *versus* ‘non-fishing’) and the counts of *Anopheles* larvae found inside individual habitats. Habitat type was further classified using bottom surface type (i.e. wooden, rocky and muddy) as a possible mediator of this association [38]. Three potential qualitative moderators of this relationship namely emergent plants [39], direct sunlight [38, 39] and fish predators [40, 41] were also assessed. Each moderator was classified into two categorical predictors of *Anopheles* larval productivity i.e. exposure versus non-exposure of habitats to direct sunlight, presence versus absence of emergent plants in habitats and presence versus absence of fish predators in the habitats. Beyond the presumed effect(s) of these variables *Anopheles* larvae were assumed to eventually emerge into adult mosquitoes through the pupal stage. These classifications of variables were superseded by diligent larval sampling.

Upon arrival at a potential mosquito larval habitat the WHO dipper was lowered gently at an angle of 45 degrees just below the water surface to allow any larvae present in the water to flow into the dipper. Sampling was done between 0900 and 1100 h. Research team members ensured that the water was calm enough and their body shadows were not cast on the surface before a dip was taken. The number of *Anopheles* larvae collected from each one of the 10 dips was summed and recorded for individual habitats. Sampling was done in areas around floating debris and edges of the habitats as most preferred sites for mosquito larvae [42]. Collected larvae were identified morphologically using taxonomic keys [43]. A sub-set of mosquitoes belonging to the *An. gambiae* complex were subjected to confirmatory DNA tests to identify them to species level [44].

### Data analysis

A logistic regression model of the form “Logit (*Anopheles* present) = β_o_ + β_1_ habitat type”, where β_o_ is the intercept of the regression line on the y-axis and β_1_ is the gradient, was fitted [45] to describe the probability of finding *Anopheles* larvae in habitats created through artisanal capture fishing. Habitat type, classified as ‘fishing habitats’ or ‘non-fishing habitats’, was the predictor variable. Habitat content i.e. presence or absence of *Anopheles* larvae in the stagnant water bodies constituted the outcome variable. A P-value of 0.05 or less denoted a significant effect of artisanal capture fishing on presence of *Anopheles* larvae in habitats. The effect of artisanal capture fishing and habitat bottom surface type on the number of *Anopheles* mosquito larvae present in habitats was modelled using generalized linear models (GLM) with a Poisson distribution and a log link function [45]. The GLM models included the effects of the moderator variables on the outcome (i.e. number of *Anopheles* mosquitoes found in the habitats). All data were analysed using version 23 of the IBM SPSS statistical package.

## Results

This study was carried out in July 2017. No rains were received during this period and the preceding 2 months. Seventy-seven out of the 100 mosquitoes subjected to molecular analysis were identified as *An. gambiae* sensu stricto. The rest of the mosquitoes were not identifiable, even after several repeats. No other *Anopheles* mosquito species were identified.

### Artisanal capture fishing and creation of *Anopheles* larval habitats

#### Habitat types

**Fig. 2:**
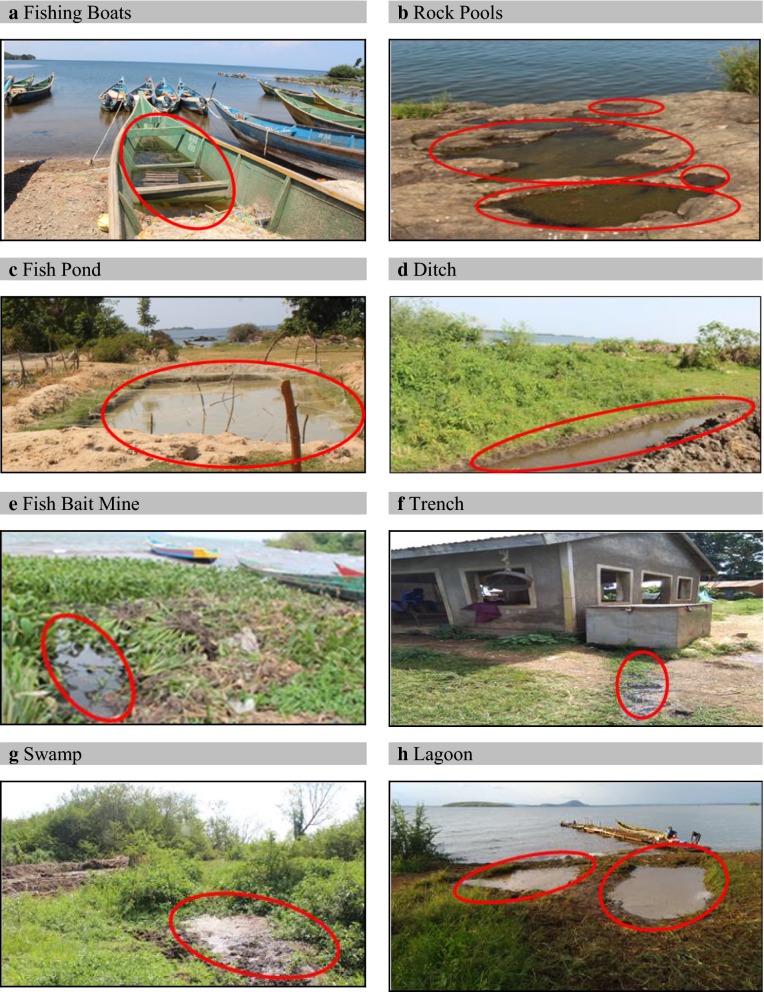
Mosquito larval habitat types found on Mageta Island in Lake Victoria, Western Kenya. Areas with stagnant water are circled in red

**Fig. 3:**
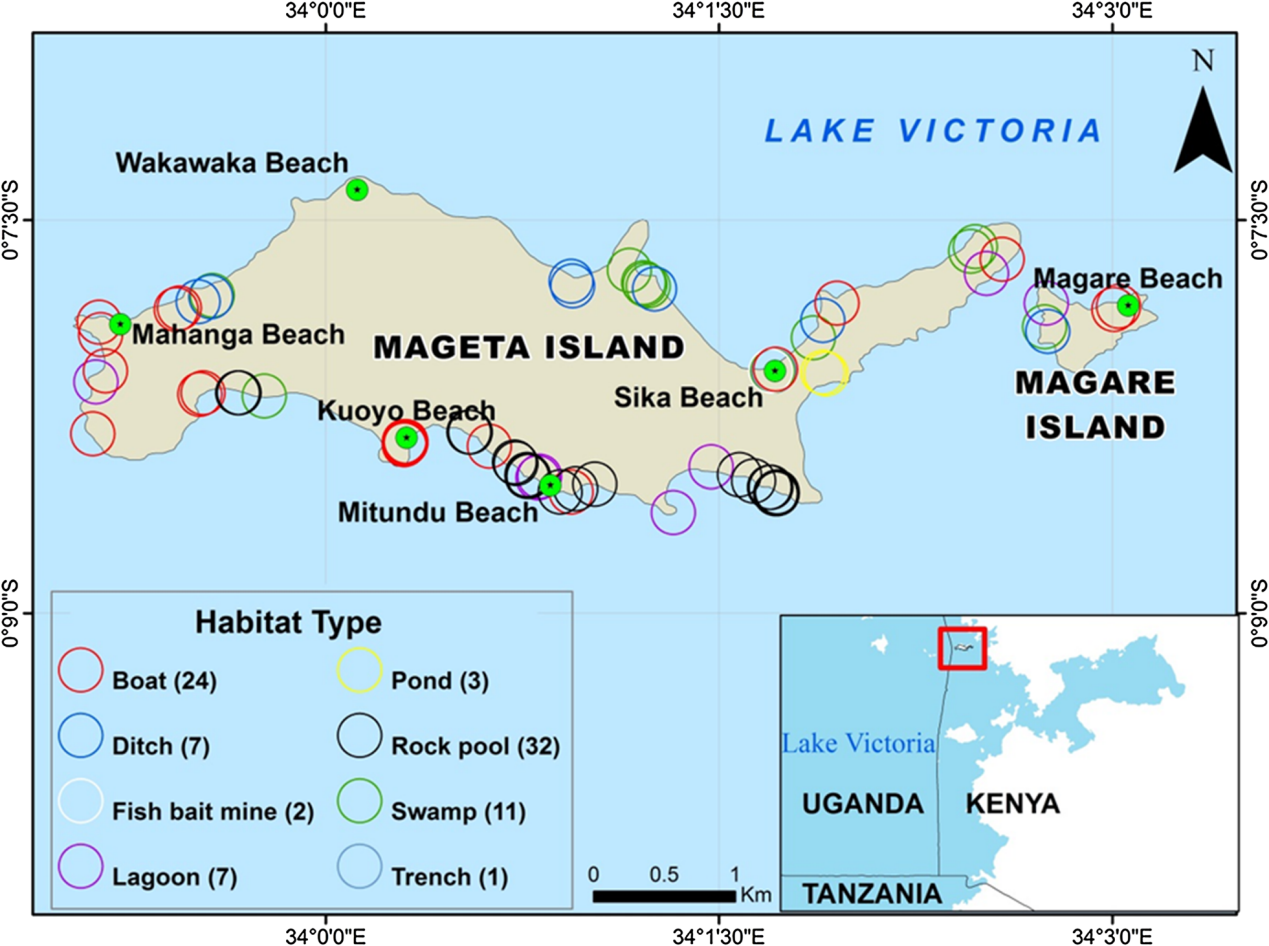
Spot map showing geographical location of *Anopheles* larval habitats on Mageta Island in western Kenya. The center of the rings is the exact location of the habitats

A total of 87 mosquito larval habitats, 82 on Mageta Island and 5 on Magare Island, were identified. The habitats, classified into eight different habitat types, included rock pools (n = 32; 36.8%), fishing boats (n = 24; 27.6%), swamps (n = 11; 12.6%), ditches (n = 7; 8%), lagoons (n = 7; 8%), fish ponds (n = 3; 3.4%), fish bait mines (n = 2; 2.3%) and trenches (n = 1; 1.1%). Most of the habitat types were located on the eastern, western and southern shores of the Island, where most artisanal capture fishing activities were concentrated. Visual representations of these habitats and a spot map showing their distribution on Mageta Island are shown in Figs. 2 and 3, respectively. All fishing boats, fish bait mines and trenches were grouped together as ‘fishing habitats’, having been created through artisanal capture fishing activities. The ‘non-fishing habitats’ included all rock pools, swamps, lagoons, ditches and fish ponds. The percentages of fishing and non-fishing habitats were 31% (N = 27) and 69% (N = 60), respectively. Fishing boats comprised 89% (24/27) of the ‘fishing habitats’.

#### Origin of mosquito breeding habitats

Evolution of the habitats was largely associated with man’s efforts to support livelihoods. For example, at the end of each fishing round fishers customarily fetch and pour fresh lake water in fishing boats after parking them ashore. Ditches, which are normally sunk below the water table, were created to prevent access of night-grazing hippopotamuses to food crops. The ditch water was also used to irrigate crop plants and, in one special case, a ditch was used for docking a fishing boat. The three fish ponds found on Mageta Island were used to culture edible fish. Stagnant water pools associated with fish bait mines resulted from excavation of earthworms from wet soil using bare hands or removing rocks/stones from crab traps. The earthworms and crabs are used locally as fishing baits. Most temporary housing structures used for primary fish processing were connected to dug-out trenches. The trenches often contained water originating from cleaning activities or slow-melting ice blocks present in leaky, locally-made cooler boxes used for temporary storage of captured fish. The identified natural habitats were largely created through wave action on Lake Victoria. Waves deposited water near the shoreline in depressions on rocks to form rock pools, in the flat littoral zone to form swamps and behind sand bars to form lagoons [46]. In all cases adult gravid female mosquitoes laid eggs in the stagnant water bodies, which acted as larval breeding resources.

#### Presence of *Anopheles* larvae in habitats

**Fig. 4:**
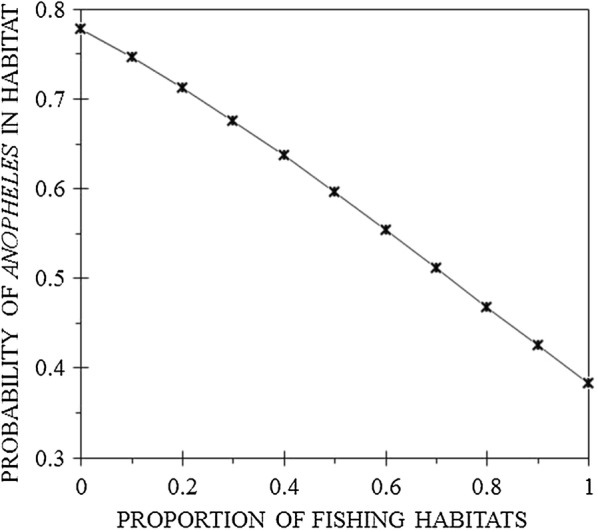
Modelled probabilities of finding *Anopheles* mosquitoes in larval habitats associated with (artisanal capture) fishing

All habitat types identified on Mageta Island with the exception of fish bait mines, fish ponds and trenches contained *Anopheles* larvae. No *Anopheles* larvae were found on Magare Island. Although one half (50.6%; 44/87) of all putative mosquito habitats contained *Anopheles* larvae, 74% (20/27) of ‘fishing habitats’ and only 38.3% (23/60) of ‘non-fishing habits’ contained *Anopheles* larvae. Eighty-three percent (20/24) of the fishing boats contained *Anopheles* larvae. Fishing boats were the only ‘fishing habitats’ that contained *Anopheles* larvae. These data underscore the importance of artisanal fishing on the epidemiology of malaria on Mageta Island. The fitted logistic regression model (χ^2^ = 12.11, df = 1, N = 87, p < 0.001) found a significant negative association between artisanal capture fishing and the probability of finding *Anopheles* larvae in the habitats (P = 0.001) (Fig. 4). The odds of finding *Anopheles* larvae in a habitat on Mageta Island decreased by 0.173 (95% CI = 0.062–0.505) for each unit increase in the proportion of habitats associated with artisanal capture fishing.

### Artisanal capture fishing and *Anopheles* larval productivity

#### Anopheles density in different habitat types

**Fig. 5:**
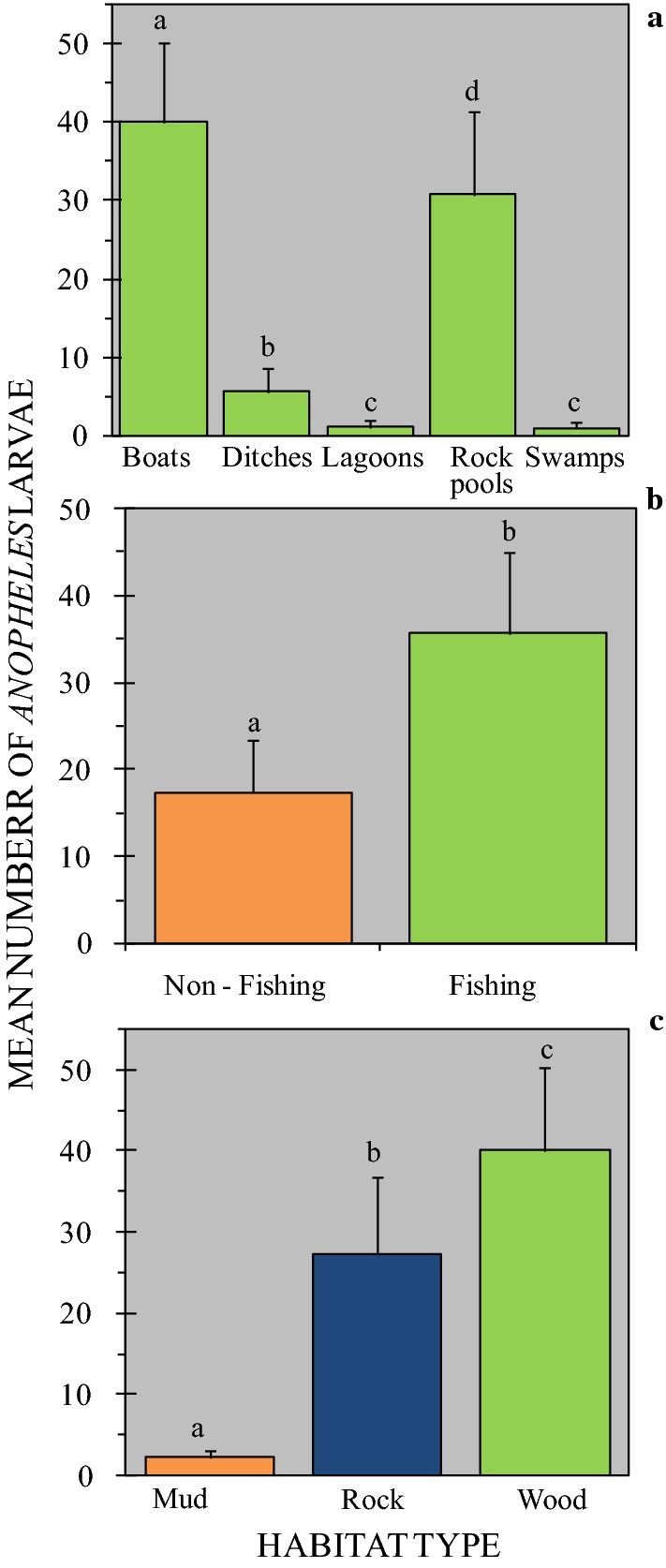
Mean number of *Anopheles* larvae collected from different mosquito habitat types (**a**) on Mageta Island in western Kenya. Mean numbers of larvae collected from ‘fishing’ versus ‘non-fishing’ habitats (**b**) and from habitats with different surface bottom types (**c**) plus the standard errors of the mean values are shown. Bars with different letters (within the same panel) denote a significant difference in the mean number of mosquitoes collected. Similar letters (within the same panel) indicate no difference in the mean number of mosquitoes collected. No *Anopheles* larvae were recovered from fishing ponds, fish bait mines and trenches

The total number of *Anopheles* larvae collected in this study was 2008, with a mean density of 23.08 ± 5.05 individuals per habitat. Forty-eight percent of the larvae were recovered from fishing boats and 49% from rock pools. Despite being the most common habitat type, the mean number of *Anopheles* larvae present in rock pools (30.81 ± 10.54) was significantly lower than those found inside fishing boats (40.08 ± 10.16) (P = 0.001). The mean number of *Anopheles* larvae in ditches, lagoons and swamps was 5.71 ± 3.11, 1.14 ± 0.9 and 1.09 ± 0.7, respectively (Fig. 5a). These data underscore the importance of fishing boats (hence artisanal capture fishing), rock pools and, to a lesser extent, ditches on the overall epidemiology of malaria on Mageta Island.

#### Anopheles density in ‘fishing’ versus ‘non-fishing’ habitats

About half (48%) of malaria mosquito larvae were recovered from ‘fishing habitats’. However, the mean *Anopheles* larval density in the ‘fishing habitats’ (35.7 ± 1.15) was significantly higher than that in ‘non-fishing habitats’ (17.4 ± 0.539) (P = 0.001) (Fig. 5b). This implies that there is a potential relationship between artisanal capture fishing and the density of *Anopheles* larvae found in habitats on Mageta Island. While no *Anopheles* larvae were found in fish bait mines and the trench, *Anopheles* eggs were observed in the fish bait mines and inside fishing boats.

#### Anopheles density in habitats of different bottom surface types

Inspection of bottom surfaces of identified habitats revealed that 27 had mud (3 lagoons, 11 swamps, 7 ditches, 2 fish bait mines, 1 trench and 3 fish ponds), 36 had rock (all rock pools and 4 lagoons) and 24 were wooden (all boats). The mean number of *Anopheles* in these habitats were 2.22 ± 0.29, 27.39 ± 0.87 and 40.08 ± 1.29, respectively (Fig. 5c). Because all fishing boats were wooden the significant association between artisanal capture fishing and *Anopheles* larval productivity is likely to have been driven by some evolutionarily beneficial aspect(s) of the timber used to construct the boats. Similarly, the high number of *Anopheles* larvae encountered in rock pools is predictive of factors associated with this habitat type that promote colonization. Pairwise comparisons revealed significant statistical differences in *Anopheles* density between mud versus rock bottomed habitats (P = 0.001), mud versus wood bottomed habitats (P = 0.001) and wood versus rock bottomed habitats (P = 0.001).

#### Effect of moderator variables on Anopheles larval productivity

The correlation between *Anopheles* larval productivity and the interaction between artisanal fishing and exposure of habitats to direct sunlight could not be determined. This was also the case for the relationship between *Anopheles* larval productivity and the interaction between artisanal fishing and presence of emergent plants/fish predators in habitats. These potential moderator variables were all declared statistically redundant given that all fishing habitats were exposed to direct sunlight, no fishing habitats had emergent plants in them and only one fishing habitat contained fish predators. The interactions of these moderating variables with artisanal fishing are not discussed further on. However, as expected from examining the main effects, exposure to direct sunlight had a significant effect on *Anopheles* larval density inside habitats [38, 39] (P = 0.001). More *Anopheles* larvae were recovered from habitats exposed to direct sunlight. Similarly, presence of emergent plants and fish predators in habitats both had significant effects on *Anopheles* larval density (P = 0.001). Habitats that had emergent plants in them and those that harbored larvivorous fish had significantly fewer *Anopheles* larvae (P = 0.001; Table 1).

**Table 1:** Mean number (± SE) of *Anopheles* larvae recovered from aquatic habitats that were non-exposed (predictor absent) and exposed (predictor present) to different moderating effects on Mageta Island in western Kenya

Predictor	N	Mean (± SE) number of *Anopheles* larvae		Exp (B)	P
		Predictor absent	Predictor present		
Emergent plants	9	25.54 ± 0.57	1.87 ± 0.48	13.621	0.001
Fish predators	22	30.85 ± 0.69	0.10 ± 0.07	323.885	0.001
Direct sunlight	73	23.43 ± 1.29	23.32 ± 0.57	1.005	0.938

The test statistic {Exp (B)} and the level of statistical significance between the mean numbers of larvae in exposed and non-exposed habitats are shown for each predictor. ‘N’ refers to the number of larval habitats (out of 87) ‘exposed’ to the predictor variable. The rest of the habitats were ‘not exposed’ to the predictor

## Discussion

This study applied an ecosystem approach to find out if artisanal capture fishing facilitates breeding of *Anopheles* larvae. Although 74% of ‘fishing habitats’ and only 38% of ‘non-fishing habits’ contained *Anopheles* larvae, there was a significant negative association between artisanal capture fishing and the probability of finding *Anopheles* larvae in the habitats. Interestingly, 83% of the fishing boats, which formed the majority of ‘fishing habitats, contained *Anopheles* larvae. Although the total numbers of *Anopheles* larvae collected were about equal, the mean density in ‘fishing habitats’ was twice that in ‘non-fishing habitats’. Forty-eight percent of the larvae were recovered from fishing boats and 49% from rock pools. Despite being the most common habitat type, the mean number of *Anopheles* larvae present in rock pools was significantly less than those found inside the wooden fishing boats. These data underscore the importance of artisanal capture fishing on the epidemiology of malaria on Mageta Island.

The significant negative association between artisanal capture fishing and the probability of finding *Anopheles* larvae in habitats is puzzling on initial thought. However, this relationship is not infinite. The fitted logistic regression equation predicts that if 100% of breeding habitats on Mageta Island were to be created through artisanal capture fishing then only 38% of them would contain *Anopheles* larvae. On the contrary over half (78%) of stagnant water bodies would contain *Anopheles* larvae if no single breeding habitat on Mageta Island was to be created through artisanal capture fishing. This analysis implies that although artisanal capture fishing is an important facet of malaria epidemiology on Mageta Island, other drivers of endemicity do exit. Thus, malaria control efforts need to be informed by holistic approaches that recognize the interdependent nature of health and other societal, developmental and ecosystem factors [25].

Peer reviewed literature about the breeding of *Anopheles* larvae in boats (or any wooden containers) is scarce. However, this was one of the most fascinating findings of this study. Traces of available data relate to the role of boats and other transport vessels as agents for the worldwide dispersal of arthropod vectors [47, 48]. What is more is that these data largely derive from observations on *Aedes,* and to a lesser extent *Culex*, species outside Africa [48–52]. Two recent studies document utilization of boats for breeding by *Anopheles coluzzii* (initially the M form of *An. gambiae* sensu lato) in two fishing communities within the Wouri river estuary near the port of Duala in Cameroon [53, 54]. Data in this article corroborate these findings, *albeit* with respect to *An. gambiae* s.s., which was the only *Anopheles* species identified on Mageta Island. Mbida et al. [54] explain the phenomenon of *An. coluzzii* breeding in boats, among other man-made habitats, as an adaptation to utilizing artificial habitats when natural ones become rare.

It is well known that *An. gambiae* uses manmade habitats for larval breeding [42, 55], but it is puzzling why fishers’ boats constituted a highly profilic *Anopheles* larval breeding resource. This study was carried out in the dry season, thus the finding that boats formed an important breeding habitat for malaria mosquitoes is confusing. The boats should have been devoid of water at this time. By iteratively engaging community actors (most of whom were artisanal fishers) it was explained that fishers engage in an active maintenance process where fresh lake water is poured in boats parked ashore between fishing rounds. The water prevents the wood from cracking. Boats not-in-use are normally stationed ashore [6, 12] during months when fishing is illegal [4, 56, 57], when fish catches are significantly low, when actors are off duty and during tumultuous party times when fishermen revel after receiving cash bonuses from their cooperative societies. That aside, it is unlikely that the larvae found in boats were introduced through the maintenance process. Strong waves must have killed any mosquito larvae present in lake water around the open beaches where fishing boats were parked. Besides the open lake is not a typical breeding habitat for *Anopheles* mosquitoes [46]. What is more is that *Anopheles* eggs, possibly introduced through direct oviposition by gravid females, were found inside the fishers’ boats.

Perhaps the dominance of *Anopheles* larvae in fishers’ boats can be best explained borrowing from life history theory [58–61]. A mosquito’s life cycle encompasses four key life history stages namely eggs, larvae, pupae and adults [62]. Eggs, larvae and pupae are aquatic, and will most likely exist in pools of water in boats for the case of malaria vectors on Mageta Island. Utilization of boats as a breeding resource is a very risky phenomenon because this habitat type is highly ephemeral. Although water is placed in the boats in the morning hours and emptied in the late afternoon on the same day or after a few days, the most common malaria vectors in the area, i.e. *An. gambiae* complex mosquitoes [32], need about 1 week to complete the aquatic cycle [63]. From an evolutionary standpoint selection pressure should favor traits that promote shorter aquatic developmental periods and production of large numbers of offspring by gravid female malaria mosquitoes. Alternatively, gravid malaria vectors may, through an ecological phenomenon referred to as ‘bet-hedging’ [60], cope by distributing single egg loads into several fishing boats containing water. Reproduction should also entail a relatively small energy investment in each offspring [58]. This should result in a sizeable number of young offspring that are capable of evading extrinsic larval mortality [59] and developing into terrestrial adult beings. The adults should then live for long enough while accessing readily available blood meals from the vast human blood meal reservoir in the fishing hamlets.

Looking further, the larger number of *Anopheles* larvae in rock pools relative to mud-bottomed habitats is not surprising. This is because larvae of *An. gambiae* s.l. are often found in habitats containing algae [39] and rock offers a better substrate for algae to grow on than mud substrates [38]. Besides, rock pools were all found near the shoreline and the water in them was frequently refreshed by spilling waves. This served to oxygenate the water, which may have promoted *Anopheles* larval productivity [64, 65]. However, the fact that most rock pools were found under tree canopies could explain the relatively lower *Anopheles* productivity compared to boats. Generally, anopheline larvae prefer open sun-lit waters [38, 39]. *Anopheles gambiae* s.l. tolerates relatively high water temperatures [65], thus the warmer sun-lit water pools in boats may have been an important factor for larval development because warm water accelerates larval development [64]. In addition, warm water temperatures in boats may have allowed more microorganisms to grow, which provided food sources for mosquito larvae [64, 66–68]. Fishing boats on Mageta Island are made using timber from the Africa teak tree (*Milicia excelsa*), commonly known as Mvule among locals. Unsubstantiated reports indicate that timber of this tree contains pores that harbour bacteria. These bacteria probably multiplied rapidly and acted as a mosquito larval food source [64, 66], so increasing *Anopheles* productivity in boats. On the contrary, presence of aged water may have harboured larger numbers of predators that suppressed abundance of *Anopheles* larvae [69] in some rock pools.

The majority of the *Anopheles* larval habitats reported in this study were created through human activities fashioned around supporting livelihoods. Artisanal capture fishing was the most notable livelihood source. This result goes in tandem with observations by other researchers in relation to crop cultivation [70, 71], livestock herding [71] and brick making [72]. The findings underscore man’s own contribution towards the viciousness of malaria and affirm the link between malaria and poverty, hence the poverty trap formed by the ecology of infectious diseases [73, 74]. This implies that the poor of the south (e.g. the artisanal fishers of Mageta Island) whose wealth, by definition, is primarily gained by extracting natural resources [19] are unable to make enough to lift themselves out of poverty [75]. They are stuck in a cycle of poverty that is almost impossible to break [73]. As fishing activities intensify so does the chance of increasing *Anopheles* larval densities in breeding habitats. This fuels malarial disease, hence the need to extract more fish to generate income as a coping strategy towards treatment.

The cross-sectional design used in this study presents several shortfalls towards associating cause to effect [76]. First, although it is generally impossible to infer the temporal sequence between exposure and outcome in cross-sectional studies, it makes biological sense, in this study, to assume that the presence of water in fishing habitats (the exposure) preceded the appearance of *Anopheles* larvae in the water (the outcome). This is because stagnant water is a prerequisite for oviposition and larval development. Second, cross-sectional studies tend to identify a high proportion of prevalent (rather than incident) cases/outcomes. By dialoguing with community actors it was learnt that boats containing water were parked ashore just for a few hours or days [6, 77]. Thus, it is unlikely that most of the breeding occurring in boats resulted from boats overstaying with water. Third, although this study was conducted in an informal occupational setting, it is highly unlikely that the effect of artisanal capture fishing on creation of *Anopheles* larval habitats was attenuated by inherent exclusion of ramshackle fishing boats from those sampled. Boats abandoned near the shorelines because of being in conditions of disrepair also contained water received from rainfall and/or spilling waves and were included in the sample. Thus, the study suffered a limited ‘*healthy worker survivor effect*’.

## Conclusions

The data presented in this article show that artisanal capture fishing is a key driver of malaria epidemiology on Mageta Island. This underscores the need for a deeper understanding of mosquito larval ecology rather than just mapping to know if breeding habitats of *Anopheles* are ‘*few*, *fixed* and *findable*’, and therefore amenable to larval source management [42]. Thus, embracing an ecosystem approach to human health (*ecohealth*) with respect to malaria can contribute towards attainment of acceptable levels of health that will enable people to realize sustainable livelihoods [70, 78]. Larval source management strategies in the global south should be cognizant of the heterogeneity in *Anopheles* breeding habitats created through livelihood activities.
